# *Leptotrichia amnionii* and the Female Reproductive Tract

**DOI:** 10.3201/eid1011.031019

**Published:** 2004-11

**Authors:** Vijay A.K.B. Gundi, Raoul Desbriere, Bernard La Scola

**Affiliations:** * Unité des Rickettsies, Marseille, France;; † Hôpital Nord, Marseille, France

**Keywords:** letter, reproductive tract, leptotrichia, 16S rRNA gene sequence

**To the Editor:** Detection of new bacteria, human complex microflora, by using 16S rRNA gene amplification and sequencing has been reported (*1*). 16S rRNA gene amplification and sequencing detected pyosalpinx, caused by *Leptotrichia amnioni*, in a patient whose samples were culture-negative.

This anaerobic gram-negative bacterium has been isolated only once before (*2*). A 41-year-old woman from the island of Comoros who had been having lower abdominal pain for 6 days was admitted to the emergency department of Hôpital Nord in Marseille. The patient's history included type 2 diabetes mellitus treated by metformin and laparoscopy to explore infertility. On examination, the patient had a pulse rate of 90 beats per min, a blood pressure of 130/80 mm Hg, and a temperature of 38.5°C. Her abdomen was not distended, but diffused lower abdominal tenderness, especially at the right iliac fossa, was present. Blood testing showed a leukocyte count of 7.7x10^9^/L, hemoglobin of 13.1g/dL, and platelet count of 213x10^9^/L. The chemistry panel showed hyperglycemia (14.1 mmol/L) and elevated C-reactive protein (254 mg/L). Renal and liver function test results were all within normal limits. Serum β-human chorionic gonadotropin was negative. A computed tomographic scan of the abdomen and pelvis showed two septated adnexal masses, a 12x7x5 cm mass on the right and a 6x4x2 cm mass on the left; the patient was referred to the gynecologic surgery department. Gynecologic examination showed greenish, purulent vaginal discharge and a fluctuant mass in the pouch of Douglas. Uterine cervical motion caused pain to the patient. Transabdominal and transvaginal ultrasound scan showed a 10x7x5 cm homogeneous liquid mass in the pouch of Douglas.

The patient was taken to the operating room and prepared for surgery. The gynecologic team performed a laparotomy that showed a 5-cm, left hydrosalpinx and a 10-cm, right tuboovarian abscess adherent to the uterus, sigmoid colon, pelvic sidewall, and pouch of Douglas. The appendix and other viscera were normal. The adhesiolysis led to the rupture of the abscess and discharge of clear greenish pus, a sample of which was sent to the laboratory for culture. Antimicrobial drug treatment was started with intravenous cefazolin, gentamicin, and metronidazole. On the first postoperative day, the patient was afebrile. Oral amoxicillin plus clavulanic acid was administered for 15 days, and oral ciprofloxacin was administered for 20 days. The patient was discharged on day 7 of hospitalization and was well at the follow-up examination 1 month later.

After Gram staining, a sample of the abscess drainage was injected onto Columbia agar with 5% sheep blood (bioMerieux, Marcy l'etoile, France) under 5% CO_2_ and anaerobic atmosphere. Antimicrobial susceptibility of the sample was tested by an agar diffusion method (*3*). A drop of the sample was deposited on an agar plate flooded with a suspension of a antimicrobial susceptible strain of *Micrococcus luteus*. After 24 h of incubation at 37°C, presence of antimicrobial activity in the sample was evident by a visible area of growth inhibition of *M. luteus* around the sample. Procedures for DNA extraction and16S rRNA gene amplification and sequencing have been detailed (*4*).

Gram staining of the sample showed numerous polymorphonuclear leukocytes and gram-negative bacteria. Culture of the sample remained sterile after 20 days of incubation, and antimicrobial susceptibility was found. The 16S rRNA gene amplification and sequencing determined a 1,493 nucleotide sequence. This sequence had 99.7% nucleotide similarity with that of *L. amnionii* (GenBank accession no. AY078425), which corresponded to a difference of 4 nucleotide. The 16S rRNA gene sequence of the detected bacterium was deposited under accession no. AY489565. *L. amnionii* was previously recovered in anaerobic culture of the amniotic fluid of a woman after intrauterine fetal demise (*2*). It was isolated on blood and chocolate agar under anaerobic conditions and showed very small gray colonies of <1 mm. This slow-growing bacterium was lost after two subcultures, and no isolate is available for further description (*2*). In that case and in the case reported here, the patients had uneventful recoveries after an amoxicillin plus clavulanic acid antimicrobial regimen was given. This bacterium and our isolate are related to, but different from, *L. sanguinegens*.

*Leptotrichia* is a small genus closely related to *Fusobacterium* and comprises slow-growing, gram-negative, filamentous, anaerobic bacterial flora of the oral cavity and genital tract (*5*). Species included in the genus are *L. buccalis*, *L. trevisanii*, *L. sanguinegens*, and *L. amnionii* (*2**,**6*). All *Leptotrichia* species are extremely fastidious and cannot be grown easily on conventional microbiologic media or by conventional methods. As evidenced by sequences available in the GenBank database, most of the 16S rRNA gene sequences are from cloned DNA from complex flora but not from bacterial isolates. *Leptotrichia* species has been suspected to play a role in periodontal disease. However, *Leptotrichia* species have only been associated with serious systemic disease, usually in immunocompromised patients (*7**,**8*). Bacteremia caused by *L. sanguinegens* in pregnant women has also been reported (*9*). More widespread use of polymerase chain reaction amplification and sequencing of the 16S rRNA gene for identification or detection of fastidious pathogens in humans will likely provide verification of several new pathogens that are now part of normal human flora.
